# Ibrutinib combinations in CLL therapy: scientific rationale and clinical results

**DOI:** 10.1038/s41408-021-00467-7

**Published:** 2021-04-29

**Authors:** Natalia Timofeeva, Varsha Gandhi

**Affiliations:** 1 Departments of Experimental Therapeutics, The University of Texas MD Anderson Cancer Center, Houston, TX 77030 USA; 2 Departments of Leukemia, The University of Texas MD Anderson Cancer Center, Houston, TX 77030 USA

**Keywords:** Combination drug therapy, Medical research

## Abstract

Ibrutinib has revolutionized the treatment of chronic lymphocytic leukemia (CLL). This drug irreversibly inhibits Bruton tyrosine kinase (BTK) by covalently binding to the C481 residue in the BTK kinase domain. BTK is a pivotal protein for B cell receptor signaling and tissue homing of CLL cells. Preclinical investigations have established the importance of the B cell receptor pathway in the maintenance and survival of normal and malignant B cells, underscoring the importance of targeting this axis for CLL. Clinical trials demonstrated overall and progression-free survival benefit with ibrutinib in multiple CLL subgroups, including patients with relapsed or refractory disease, patients with 17p deletion, elderly patients, and treatment-naïve patients. Consequently, ibrutinib was approved by the US Food and Drug Administration for newly diagnosed and relapsed disease. Ibrutinib has transformed the treatment of CLL; however, several limitations have been identified, including low complete remission rates, development of resistance, and uncommon substantial toxicities. Further, ibrutinib must be used until disease progression, which imposes a financial burden on patients and society. These limitations were the impetus for the development of ibrutinib combinations. Four strategies have been tested in recent years: combinations of ibrutinib with immunotherapy, chemoimmunotherapy, cell therapy, and other targeted therapy. Here, we review the scientific rationale for and clinical outcome of each strategy. Among these strategies, ibrutinib with targeted agent venetoclax results in high complete response rates and, importantly, high rates of undetectable minimal residual disease. Although we concentrate here on ibrutinib, similar combinations are expected or ongoing with acalabrutinib, tirabrutinib, and zanubrutinib, second-generation BTK inhibitors. Future investigations will focus on the feasibility of discontinuing ibrutinib combinations after a defined time; the therapeutic benefit of adding a third agent to ibrutinib-containing combinations; and profiling of resistant clones that develop after combination treatment. A new standard of care for CLL is expected to emerge from these investigations.

## Introduction

B cell receptor (BCR) signaling is essential for B cell development and maturation. Bruton tyrosine kinase (BTK) is a critical enzyme in the BCR signaling cascade. BTK is activated by upstream Src-family kinase members (Blk, Lyn, and Fyn) and Syk kinase. Active BTK signals through further phosphorylation and activation of phospholipase Cγ2 (PLCG2) accompanied by Ca2+ mobilization. Stimulation of this pathway ultimately leads to activation of NF-κB and MAP kinase pathways, which in turn results in increased proliferation, survival, and migration of B cells^1^.

Ibrutinib is a first-in-class oral irreversible inhibitor of BTK indicated for the treatment of chronic lymphocytic leukemia (CLL) and small lymphocytic lymphoma in adults. Ibrutinib has transformed the landscape of CLL treatment, and the introduction of ibrutinib for CLL treatment marked the beginning of the era of kinase-targeted drugs in this disease. Following ibrutinib’s success, other BTK inhibitors were developed. Three novel agents, acalabrutinib (ACP-196)^2^, tirabrutinib (ONO/GS-4059)^3^, and zanubrutinib (BGB-3111)^4^, are being widely tested for CLL patients. However, ibrutinib remains the most well studied and predictable BTK inhibitor at present^5^.

## Clinical trials leading to ibrutinib approval

The efficacy and tolerability of ibrutinib were initially demonstrated in a phase I study, which showed an overall response (OR) rate of 60% and a complete remission (CR) rate of 16% in patients with relapsed/refractory CLL (Supplementary Fig. 1)^6^. Subsequent phase Ib and II studies showed OR rates of 88% and 71%, respectively, and durable remissions with manageable toxicities in patients with relapsed/refractory CLL^7^ and treatment-naïve elderly patients with CLL^8^.

The RESONATE series of randomized trials followed. In the RESONATE^9^ and RESONATE-2^10^ phase III trials, ibrutinib was compared with the anti-CD20 antibody ofatumumab and with chlorambucil, respectively. In both trials, ibrutinib resulted in a significantly higher OR rate, better progression-free survival (PFS), and better overall survival (OS). RESONATE-17, a phase II trial, showed that even in patients with relapsed/refractory CLL with deletion of 17p [del(17p)], ibrutinib represented a clinical advance, and as a result, the drug has been incorporated into treatment algorithms as a primary option for all subsets of CLL patients^11^.

## Limitations of single-agent ibrutinib

Unfortunately, single-agent ibrutinib has important limitations, including a low CR rate; resistance development due to *BTK* and other BCR pathway mutations; the risk of off-target toxic effects; and the need for long-term use and associated high cost. These constraints have led to interest in combining ibrutinib with other agents.

### Low rate of CR

Despite the high OR rate, most responses to continuous treatment with ibrutinib are partial. Fewer than 5% of all patients on ibrutinib monotherapy achieve a CR, although higher CR rates have been reported with prolonged use of ibrutinib: 8% at 27.6 months^11^, 9% at 42 months^12^, and 14% at 60 months^13^. CR achievement with ibrutinib is associated with longer PFS in CLL^14^; thus, increasing the depth of response using combination strategies could be an option to improve survival outcomes in CLL.

### Disease progression and resistance

Ibrutinib produces durable responses in most patients with CLL, but a significant proportion of patients treated with single-agent ibrutinib experienced CLL progression: 15.5% of treatment-naïve patients and 20.9% of patients with relapsed/refractory disease^13,15^. In ibrutinib-resistant CLL, the phenomenon of clonal evolution with the development and selection of resistant clones has been reported^16^. Several groups have identified that in the BCR pathway, resistant clones acquire C481 *BTK* domain mutations^16,17^, exhibit alterations in downstream molecules^16,18^, or become BCR signaling independent^18^. The most commonly studied mechanisms of resistance to ibrutinib are mutations in *BTK* and *PLCG2*^19^, which were found in 85% of patients at the time of CLL progression^19^. The cumulative incidence of CLL progression was estimated to be 0.7% at 1 year and 19.1% at 4 years of ibrutinib therapy^19^. One approach to mitigate the development of resistance-associated mutations is to combine ibrutinib with agents targeting other aspects of CLL pathophysiology.

### Toxicity

In addition to inhibiting BTK, ibrutinib inhibits multiple other kinases, including EGFR, TEC, IL-2-inducible T cell kinase (ITK), and TXK, and off-target inhibition appears to contribute to untoward toxicities^20^ necessitating dose reduction or discontinuation^13,21^. Although O’Brien et al.^13^ found that the toxicity of ibrutinib decreased with time, ibrutinib-related adverse events (AEs) continued to occur even after years of ibrutinib therapy^13^. Fifteen patients discontinued ibrutinib by 3 years and 34 discontinued ibrutinib by 5 years due to AEs. Similarly, 22 patients discontinued ibrutinib by 3 years and 35 discontinued ibrutinib by 5 years due to disease progression. An increase in the number of AEs was observed in the RESONATE and RESONATE-2 trials when data were compared between initial reporting at 9.4 months and later reporting at 19 months^22^.

### Financial burden on patients and society

At the dose of 420 mg daily, the cost of ibrutinib therapy in the USA is approximately US$130,000 per year, and patients are on ibrutinib until disease progression or unacceptable toxicity. In contrast, the price of six cycles of chemoimmunotherapy ranges from US$45,000 to US$100,000, indicating that ibrutinib adds a significant burden to private or government payers and patients^23^. With ibrutinib-based combination strategies, it may be possible to discontinue therapy at some point.

## Ibrutinib combinations

Four different ibrutinib combinations have been tested in recent years: combinations with immunotherapy, chemoimmunotherapy, cell therapy, and other targeted therapy.

### Ibrutinib and Immunotherapy

#### Scientific rationale

**Fig. 1:**
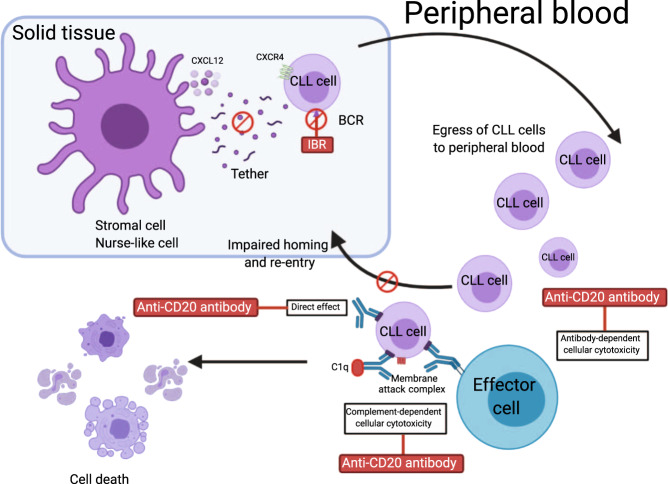
Rationale for ibrutinib combination with monoclonal antibodies. Ibrutinib (IBR) reduces levels of smCXCR4, leading to enhanced CLL cell egress from the lymph nodes and spleen to the circulation and impaired homing to the nodal compartments. In peripheral blood, CLL cells are exposed to monoclonal antibodies by three major independent mechanisms: (1) antibody-dependent cellular cytotoxicity (ADCC), (2) complement-mediated cytotoxicity, and (3) direct apoptosis. This figure was created with BioRender.com. BCR, B cell receptor.

A study of mechanisms of CLL lymphocytes’ retention in niches showed that inhibition of BTK by ibrutinib reduced cell surface levels of CXCR4 receptor. Decreased surface membrane levels of CXCR4 in turn aborted cycling from and to the membrane, which resulted in rapid redistribution of CLL cells from spleen and lymph nodes into the circulation^24^. Most patients treated with ibrutinib experience lymphocytosis due to lymphocyte egress from nodal compartments^25^. It was proposed that combining ibrutinib with anti-CD20 therapy will target and clear blood lymphocytes and shorten the time to response by reducing the duration and incidence of redistribution lymphocytosis^26^ (Fig. 1). Conversely, preclinical studies suggested potential antagonistic effects of ibrutinib combined with anti-CD20 monoclonal antibodies due to off-target activity against ITK. ITK inhibition by ibrutinib impairs NK cell function and decreases the efficacy of antibody-dependent cellular cytotoxicity (ADCC)^27^. Additionally, ibrutinib reduces phagocytosis of rituximab-coated leukemic cells by macrophages or neutrophils^28^, and ibrutinib strongly inhibited all cell-mediated mechanisms induced by rituximab, ofatumumab, or obinutuzumab^27^. Compared to rituximab and ublituximab, ofatumumab has a greater dependence on complement-dependent cytotoxicity, which stays well maintained during ibrutinib therapy^29,30^. Obinutuzumab utilizes alternative pathways to ADCC and has a higher programmed cell death efficacy than rituximab. Thus, ofatumumab and obinutuzumab seem to be superior to rituximab and ublituximab for combination with ibrutinib, which has an adverse impact on ADCC and phagocytosis^29^.

#### Clinical results

Based on the above scientific rationale for combining ibrutinib with anti-CD20 therapy, rituximab, ofatumumab, obinutuzumab, and ublituximab have been combined with ibrutinib (Supplementary Table 1). Concurrent administration of ibrutinib and rituximab was tested in 40 patients with CLL (*n* = 36 previously treated) with high-risk disease^31^. The OR rate was 95%; however, most of the responses were partial responses (87%). At 18 months, the estimated PFS rate was 78%, and the estimated OS rate was 84%.

Later, in a head-to-head comparison, 208 patients with CLL were assigned to ibrutinib or ibrutinib plus rituximab^32^. Combination therapy did not produce a higher CR rate than single-agent ibrutinib in patients with relapsed/refractory disease (*p* = 0.32) but did produce a higher CR rate in patients with del(17p) and/or *TP53* mutation and treatment-naïve patients. The PFS rates at 3 years were similar, indicating that adding rituximab did not improve survival. However, the level of peripheral blood lymphocytes normalized faster and CR was achieved earlier in patients in the combination therapy group. Bone marrow minimal residual disease (MRD) levels at 12 months were lower in patients receiving the combination (18.5% vs 34.4%, *p* < 0.0001)^32^.

In the ALLIANCE trial, bendamustine plus rituximab (BR), ibrutinib plus rituximab, and ibrutinib monotherapy were compared in 547 patients with untreated CLL. Ibrutinib-containing therapy showed a major PFS benefit compared with the BR regimen. There was no difference in the PFS rate at 2 years between ibrutinib plus rituximab and ibrutinib monotherapy (88% and 87%, respectively, *p* = 0.49). Thus, in this study, the ibrutinib plus rituximab combination showed no benefit in any clinically relevant endpoint over single-agent ibrutinib. The percentage of patients with CR and undetectable MRD was greater with BR treatment than with the ibrutinib-containing regimens^33^.

Following the ALLIANCE trial, the ECOG-E1912 trial examined the efficacy of ibrutinib plus rituximab compared with the standard six cycles of fludarabine, cyclophosphamide, and rituximab (FCR) in treatment-naïve patients with CLL. At 3 years, the PFS rate was higher for ibrutinib plus rituximab than for FCR (89.4% vs 72.9%, *p* < 0.001). But in terms of achieving CR and undetectable MRD, chemotherapy had an advantage. Patients in the ibrutinib plus rituximab group achieved CR in 17.2% versus 30.2% in the chemoimmunotherapy group. At the 12-month response assessment, the proportion of patients with undetectable MRD was dramatically lower with ibrutinib plus rituximab than with FCR (8.3% vs 59.2%)^34^.

According to the ALLIANCE and ECOG-E1912 trials, ibrutinib plus rituximab was superior to the BR and FCR regimens in terms of survival outcomes^33,34^. However, head-to-head comparison of ibrutinib plus rituximab versus ibrutinib alone showed no benefits of rituximab addition in terms of clinically significant endpoints such as PFS and OS^32^. The differences in depth of remission and time to resolution of lymphocytosis may not be sufficient to justify adding rituximab to ibrutinib.

Antagonistic cellular interactions between rituximab and ibrutinib were hypothesized to explain why clinical outcomes seemed roughly equivalent to those with ibrutinib monotherapy. Thus, ofatumumab and obinutuzumab became the next agents to be tested with ibrutinib.

A single-arm trial of ibrutinib plus ofatumumab included 66 pretreated patients with CLL or small lymphocytic lymphoma. Among all patients with CLL or small lymphocytic lymphoma, the OR rate was 83.3%. For the entire study population, the estimated 12-month PFS and OS rates were 83.1% and 88.6%, respectively. However, the CR rate was low; only one patient (1.5%) achieved a CR as the best response^26^.

In the iLLUMINATE trial, 229 patients with CLL (65% with high-risk disease) were randomly assigned to receive ibrutinib plus obinutuzumab (*n* = 113) or chlorambucil plus obinutuzumab (*n* = 116)^35^. The ibrutinib plus obinutuzumab arm had a significantly higher PFS rate (79% vs 36%, *p* < 0.0001), OR rate (91% vs. 81%), and CR rate (41% vs 16%). Importantly, the rate of undetectable MRD in bone marrow or peripheral blood was higher in the ibrutinib plus obinutuzumab group than in the obinutuzumab plus chlorambucil group (35% vs 25%)^35^.

Combination treatment gave an option to increase the depth of remission on ibrutinib and formed the concept of MRD based discontinuation of treatment. Patients with undetectable MRD become potential candidates for fixed-duration therapy with novel agents that usually must be given until disease progression.

The MRD-driven strategy was applied in the ICLL7 FILO trial of the combination of ibrutinib and obinutuzumab. Treatment-naïve patients with CLL were enrolled to receive nine cycles of this combination as induction therapy. Patients with CR and undetectable MRD continued ibrutinib monotherapy for 6 months; other patients received four cycles of fludarabine, cyclophosphamide, and obinutuzumab along with ibrutinib (iFCG). After induction therapy, among 130 patients, only 10 (8%) had an MRD-negative CR and thus were eligible for switching to ibrutinib monotherapy. The remaining 120 patients were assigned to receive iFCG. At the final MRD evaluation point after both steps of treatment, 73% of patients had a CR and 79% had undetectable MRD in the bone marrow. The addition of chemotherapy played a significant role in attaining deeper responses. The toxicity remained stable throughout the two phases, and no unpredictable AEs were registered^36^.

The fourth anti-CD20 monoclonal antibody, ublituximab, was studied in combination with ibrutinib in two clinical trials. Sharman et al. reported that ibrutinib plus ublituximab produced an OR rate of 90% in 41 patients^37^ in a phase II trial; this group also reported that preliminary results showed an advantage of ibrutinib plus ublituximab over single-agent ibrutinib in terms of OR rate (80% vs 47%, *p* < 0.001) in a randomized phase III trial^38^.

In summary, there are some successful examples of adding an anti-CD20 monoclonal antibody to ibrutinib therapy. However, the improvements in survival outcomes did not represent as dramatic a breakthrough in CLL therapy as did adding an anti-CD20 antibody to chemoimmunotherapy regimens.

### Ibrutinib and Chemoimmunotherapy

Before the introduction of ibrutinib, chemoimmunotherapy regimens, such as FCR^39^ and BR^40^, were standard of care and widely used for patients with CLL. FCR was considered the gold-standard first-line therapy for physically fit young patients with CLL^39,41,42^, and BR was an option for elderly patients with comorbidities^40,43,44^. Unfortunately, patients with del(17p) or *TP53* mutations respond poorly to chemoimmunotherapy and typically have short remissions^45^. Chemoimmunotherapy-induced undetectable MRD has been associated with long-term disease-free survival, especially in patients with mutated IGHV^46,47^. However, chemoimmunotherapy is also associated with significant immediate and late complications, including a relatively high rate of therapy-related acute myeloid leukemia/myelodysplastic syndrome after front-line FCR-based regimens^39,48^.

#### Scientific rationale

**Fig. 2:**
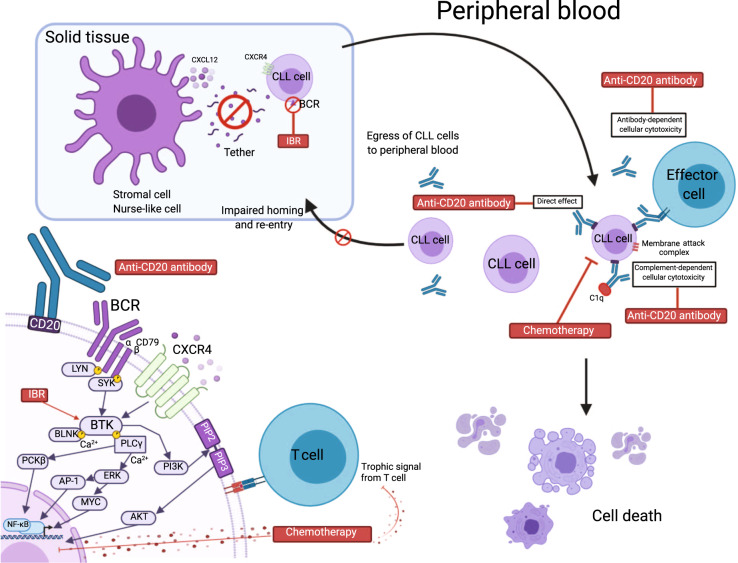
Rationale for ibrutinib combination with chemoimmunotherapy. Ibrutinib (IBR) leads to CLL cell mobilization from the nodal compartment, making CLL cells more accessible to chemotherapy and anti-CD20 antibody in peripheral blood. Lower left diagram depicts intracellular mechanism of combination. Anti-CD20 antibody along with chemotherapy induces apoptosis and immunologic cell death, while ibrutinib affects proliferation, survival, and migration of B cells. Chemotherapeutic agents such as fludarabine are well known to cause depletion of all T cell subsets, thereby reducing negative trophic signals between T cells and CLL cells. This figure was created with BioRender.com. BCR, B cell receptor.

The scientific rationale for adding ibrutinib to chemoimmunotherapy is based on a number of observations (Fig. 2). First, CLL cells reside in peripheral blood, bone marrow, and lymph nodes. Chemoimmunotherapy effectively eradicates disease in blood and bone marrow but has little impact on disease in lymph nodes^49^. The addition of ibrutinib could mobilize CLL cells from lymph nodes into the blood to make them susceptible to chemotherapy. Second, CLL cells benefit from interactions with the microenvironment, especially T lymphocytes. Studies in animal models have demonstrated a direct correlation between T cell levels in mouse blood and leukemic cell proliferation^50,51^. Interaction between CLL cells and T cells may drive T cells towards an anergic state, thereby facilitating tumor escape from immune surveillance^52^. Fludarabine-based regimens induce a severe and prolonged depletion of T cell subsets, and studies have documented sensitivity of CD4 and CD8 T cells to fludarabine^47^. Thus, T cells that interact with CLL cells could be removed by fludarabine, complementing the inhibitory effect of ibrutinib on BCR signaling. Third, ibrutinib has been shown to boost the CD8 population and may exert antitumor action through effects on CLL T cells^52^.

#### Clinical results

Several investigators have tested combinations of ibrutinib with chemoimmunotherapy (Supplementary Table 2). In one study, ibrutinib in combination with six standard cycles of BR (30 patients) or FCR (three patients) was tested^53^. For BR plus ibrutinib, the OR rate was 96.7%, and the CR rate was 40%; the estimated PFS rates were 86.3% at 12 months, 78.6% at 18 and 24 months, and 70.3% at 36 months.

Prior promising results of a phase II study of BR (NCT00274989)^40^ and the above-mentioned study of BR plus ibrutinib^53^ provided the rationale for the HELIOS study^54^. In this study, 578 patients with relapsed/refractory CLL without del(17p) received BR for a maximum of six cycles and were then randomized 1:1 to receive either placebo or ibrutinib. The addition of ibrutinib to BR significantly improved OR (*p* < 0.0001), PFS (*p* < 0.0001), and CR rate. At 17-month median follow-up, the proportion of patients with undetectable MRD was 13% in the ibrutinib group and 5% in the placebo group (*p* = 0.0011). For patients receiving ibrutinib, the undetectable MRD rate tended to increase over time and reached 26.3% by 36 months^55^. The addition of ibrutinib did not increase the rate of AEs^54^.

The FCR regimen has exhibited good efficacy as a front-line treatment for low-risk young, fit patients with CLL^41^. According to the results of a trial of FCR at The University of Texas MD Anderson Cancer Center, the PFS rate was approximately 55% after a follow-up period of 10 years for patients with mutated IGHV. A plateau was reached after 8 years, suggesting that FCR may cure patients with mutated IGHV^46^. To further improve upon these results and target lymph node-resident CLL cells, ibrutinib was added to fludarabine-and-cyclophosphamide-containing regimens for treatment-naïve CLL.

In the first study, ibrutinib was combined with FCR. In this study, 85 previously untreated patients ≤65 years of age were enrolled; 58% of the patients had unmutated IGHV. Starting 7 days after initiation of ibrutinib, ibrutinib plus FCR was started and was continued for up to six cycles. After 2 years, patients with undetectable MRD in the bone marrow had the option to discontinue ibrutinib. The rate of CR with undetectable MRD in the bone marrow 2 months after ibrutinib plus FCR was 33%, compared to the historical rate of 20% with FCR. The rate of CR or CR with incomplete hematologic recovery (CRi) increased over the course of treatment. Overall, 83.5% of patients achieved undetectable MRD in the bone marrow as best response. At a median follow-up time of 11.3 months after ibrutinib discontinuation, patients with undetectable MRD in bone marrow maintained this status according to tests for MRD in blood. These findings appear promising, especially with a favorable safety profile^56^, although longer follow-up and more data are required to evaluate the role of ibrutinib plus FCR as a front-line treatment in younger patients with CLL.

In the second study of ibrutinib plus fludarabine-and-cyclophosphamide-containing regimens, ibrutinib was combined with three cycles of fludarabine, cyclophosphamide, and obinutuzumab (iFCG) in patients with IGHV-mutated CLL^57^. It is assumed that reducing the amount of chemotherapy might lower the risk of therapy-related acute myeloid leukemia/myelodysplastic syndrome. Thus, in patients with undetectable MRD after 12 cycles, all therapy was stopped, including ibrutinib. Forty-five patients were treated. After three cycles of iFCG, 39% of patients achieved a CR/CRi, and 89% achieved undetectable MRD in the bone marrow. After 12 cycles, 73% of patients achieved a CR/CRi, and 100% achieved undetectable MRD in the bone marrow. Most patients (*n* = 41) completed all planned cycles of treatment, and all patients discontinued ibrutinib since all achieved undetectable MRD. No patient had MRD recurrence, CLL progression, or Richter transformation, with a median follow-up of 18.7 months after discontinuation of ibrutinib. The most common grade 3-4 AEs were hematological.

These two investigations^56,57^ showed promising results for time-limited treatment with combinations of ibrutinib and chemoimmunotherapy.

### Ibrutinib and cell therapy

Chimeric antigen receptor T cell (CAR-T) immunotherapy is at an early stage, but combinations of ibrutinib with CAR-T cells may be an option for patients with CLL with an unfavorable prognosis^58^.

#### Scientific rationale

Several treatments commonly used for CLL, including fludarabine, bendamustine, and alemtuzumab, adversely affect T cell function and intensify the T cell defect in CLL^47,59,60^. In contrast, ibrutinib has minor or no negative effects on the T cell compartment and has the potential to improve antitumor T cell immunity^61^. An off-target effect of ibrutinib is that it affects ITK, which shifts T cells towards a Th1 cytokine secretion profile^62^. Therefore, before and after autologous T cell collection, ibrutinib may redirect the immune response of T cells from a Th2 profile to a Th1 profile. Th1 shift is more favorable for proliferation and maintenance of chimeric receptor-expressing T cell populations^61,63^.

**Fig. 3:**
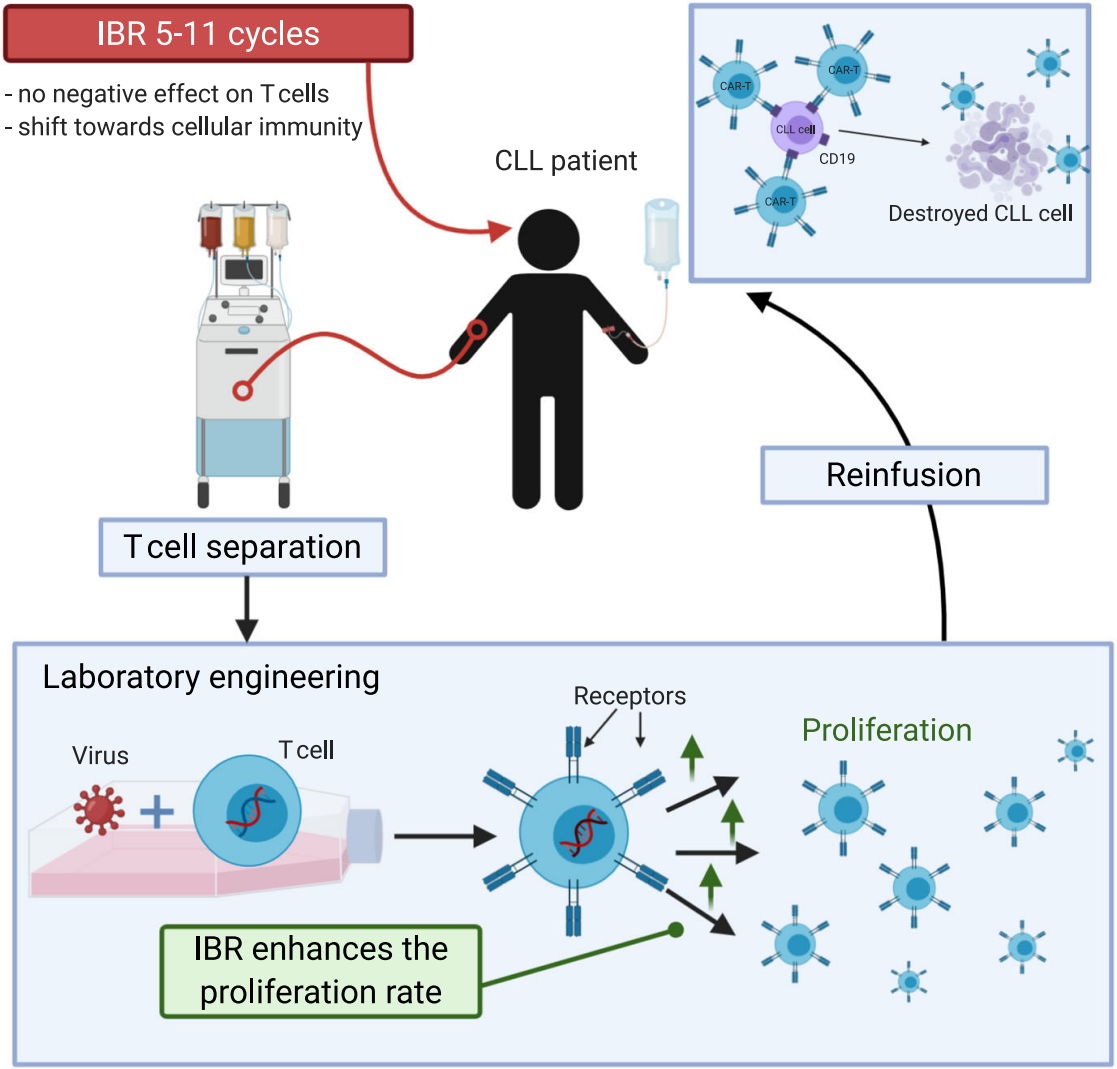
Rationale for ibrutinib combination with cell therapy. Unlike several commonly used treatments (chemotherapy, alemtuzumab), ibrutinib (IBR) does not have a profound negative impact on T cell function. Due to off-target effect of ibrutinib on ITK, there is a shift towards cellular immunity (Th1 ↑, Th2 ↓). Ibrutinib therapy for five to 11 cycles before T cell separation leads to an increased CAR-T cell proliferation rate. The mobilizing effect of ibrutinib may make CLL cells more accessible to CAR-T cells. This figure was created with BioRender.com.

One of the important requirements for effective T cell-based cancer immunotherapy is ability of T cells to effectively traffic to the tumor microenvironment. The presence of bulky disease in patients with CLL leads to reduced T cell infiltration of nodal compartments and therefore decreased antitumor activity^64^. The mobilizing effect of ibrutinib actually triggers egress of CLL cells to peripheral blood, potentially making them more accessible to CTL019 cells^65^. Thus, ibrutinib may improve outcomes in patients with CLL receiving CAR-T cells, providing a rationale to combine ibrutinib with CAR-T cell therapy (Fig. 3).

#### Clinical results

Most of the trials of ibrutinib in combination with CAR-T cell therapy have included only small cohorts of patients, but the trials show some benefit of ibrutinib plus CAR-T cells for patients with a poor prognosis. Fraietta et al.^63^ reported their experience in three patients who were treated with ibrutinib monotherapy for at least 1 year before autologous T cell collection for CAR-T treatment. The proliferative potential of patients’ T cells was observed during ibrutinib therapy. Compared to baseline results, restoration of T cell activity was detected after five to 11 cycles of ibrutinib. Prolonged ibrutinib therapy decreased the level of immunosuppressive PD-1 receptor and CD200 expression related to the functional impairment of CD8+ T cell responses. In vivo and ex vivo effects of ibrutinib positively correlated with clinical efficacy of subsequent CAR-T cell therapy. All three patients had objective responses with no evidence of lymphodepletion, and one patient had a CR. The authors concluded that concurrent administration of ibrutinib does not impair T cell activity and improves CAR-T cell effects.

The efficacy of CAR-T cell immunotherapy was also investigated by Turtle et al.^66^ in 24 patients with relapsed/refractory high-risk CLL who had received prior ibrutinib therapy. Despite a poor prognosis, the estimated OR rate was 74%, and the estimated CR rate was 21%. Seven patients (58%) had no malignant IGH clone detected. This high rate of elimination of the malignant IGH sequence from bone marrow was associated with 100% PFS and OS rates at the median follow-up of 6.6 months. CR of bulky nodal disease was less common than CR of disease in bone marrow, supporting the theory that the nodal environment has an impact on CAR-T cell infiltration and function. In this context, the mobilizing effect of ibrutinib seems to improve CAR-T immunotherapy results^66^.

Later, Gauthier et al. presented results of 19 patients treated with concurrent ibrutinib and CD19 CAR-T cells. Results were compared to those in 30 patients who received CAR-T cells without ibrutinib. The trial was focused on the feasibility and safety of the concomitant administration of ibrutinib throughout CD19 CAR-T cell therapy. In the combination treatment group, at an early time point (4 weeks), the OR rate was 83%, and the CR rate was 21% (4 of 19 patients). Sixty-one percent of the patients had undetectable MRD evaluated by IGH sequencing. There was no significant difference in 1-year PFS (*p* = 0.91) between the groups treated with and without ibrutinib. Importantly, CLL patients treated with CAR-T cells without ibrutinib experienced higher cytokine release syndrome severity and higher serum concentrations of cytokine release syndrome-associated cytokines than did patients treated with CAR-T cells with concurrent ibrutinib, despite equivalent CAR-T cell expansion in vivo^67^.

TRANSCEND CLL 004 was a phase I trial of a novel CAR-T cell product, lisocabtagene maraleucel (liso-cel). Forty-two patients with CLL were assigned to receive liso-cel as monotherapy (*n* = 23) or in combination with ibrutinib for at least 90 days after liso-cel infusion (*n* = 19). Patients in the group treated with ibrutinib plus liso-cel were heavily pretreated and had relapsed/refractory disease after prior ibrutinib therapy. However, the OR rate after 4 weeks of treatment was 95%, and 47% of the patients (*n* = 9) had a CR/CRi. Fifteen of 18 patients (83%) still exhibited CR/CRi at the 3-month follow-up. High proportions of patients had undetectable MRD by flow cytometry (89%) and next-generation sequencing (79%). Comparison of results with those in the liso-cel monotherapy group, which is forthcoming, will define the benefit of adding ibrutinib^68^.

The success of CAR-T cell therapy in patients with CLL has bolstered an interest in CAR-NK cell therapy. Liu et al. recently reported a newer approach of using CAR-NK cells for B cell diseases, including CLL. Five patients with CLL were enrolled (including two who had Richter transformation or accelerated CLL) with high-risk genetic characteristics and a history of disease progression while receiving ibrutinib. Three patients had a CR during the first month after CAR-NK cell infusion. The patient with Richter transformation had a CR confirmed by positron emission tomography scan; however, bone marrow infiltration by CLL was observed. In contrast with CAR-T cells, allogeneic CAR-NK cells can be delivered in adoptive transfer without the risk of serious cytokine release syndrome, neurologic toxic effects, or graft-versus-host disease^69^.

Cell therapies have produced excellent responses in some patients with CLL. Larger studies comparing cell therapies with standard therapies are needed to better understand the benefits of cell therapies and how best to manage related AEs.

### Ibrutinib and other targeted therapy

In addition to BTK inhibitors, such as ibrutinib, two other kinds of targeted drugs have been approved by the US Food and Drug Administration for the treatment of CLL: a BCL-2 inhibitor (venetoclax) and PI3K inhibitors (idelalisib and duvelisib).

#### Scientific rationale

**Fig. 4:**
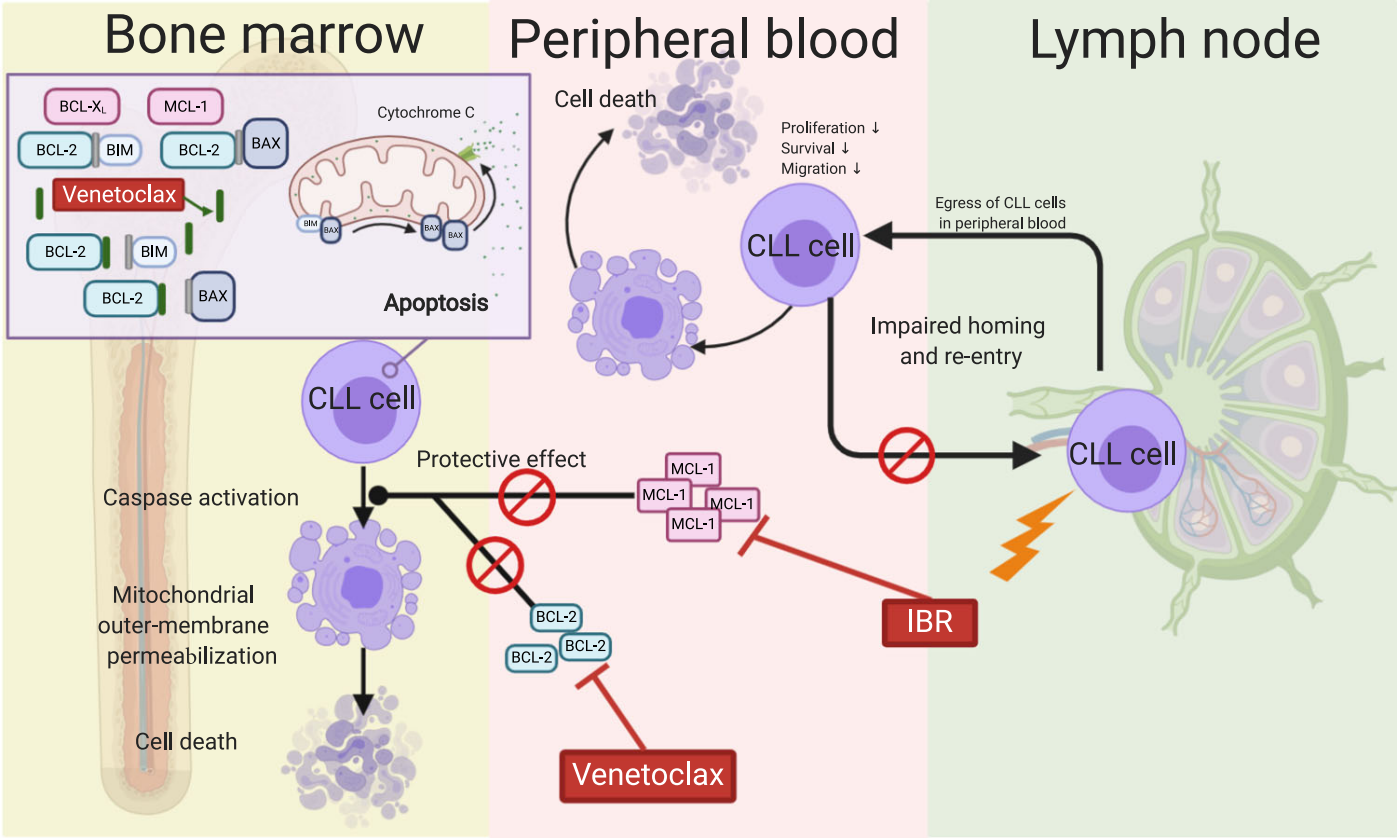
Rationale for ibrutinib combination with targeted agent venetoclax. Ibrutinib (IBR) results in mobilization of CLL cells from protective microenvironment niches in lymph nodes, while venetoclax hits CLL cells residing in the blood and bone marrow. Venetoclax targets BCL-2 to induce apoptosis, but MCL-1 could protect malignant cells from mitochondria-mediated cell death. IBR treatment results in a decrease in MCL-1 protein level and leads to synergy when combined with venetoclax. This figure was created with BioRender.com.

Since the discovery of loss of microRNAs and overexpression of BCL-2 family members in CLL lymphocytes^70^, it has been proposed that the relentless accumulation of CLL lymphocytes in the body is due to the presence of anti-apoptotic proteins of the BCL-2 family. Hence, targeting BCL-2 along with targeting the BCR pathway with ibrutinib appears to be a promising approach to target the pathophysiology of CLL. Further, ibrutinib has been shown to tackle CLL in lymph nodes, while BCL-2 inhibitor venetoclax targets CLL residing in the blood and bone marrow. Finally, ex vivo investigations during ibrutinib monotherapy demonstrated beneficial increase in apoptosis with the addition of venetoclax. Molecular mechanisms indicated decline in MCL-1 protein caused by ibrutinib^71^. Mouse models further substantiated efficacy of the combination^72,73^. Thus, the combination of ibrutinib and venetoclax seems promising on the basis of clinically complementary activity, preclinical evidence of synergism, and nonoverlapping toxicities (Fig. 4).

BTK inhibitors and PI3Kδ or PI3Kδγ inhibitors target BCR signaling through different mechanisms, and concurrent inhibition may reduce activation of escape pathways responsible for ibrutinib resistance^74^. The concept of dual BCR pathway blockade was investigated in preclinical studies. Preliminary data show that ibrutinib and PI3Kδ inhibitor idelalisib synergistically inhibit BCR-controlled adhesion, resulting in increased mobilization of CLL cells from niches^75^. Due to overlapping toxicity profiles between ibrutinib and PI3K inhibitors, the new generation of BTK inhibitors seem to be better partnered with PI3K inhibitors.

#### Clinical results

Several studies have been conducted of the ibrutinib-venetoclax combination, and some trials additionally used anti-CD20 antibody. None of the US Food and Drug Administration-approved PI3K inhibitors have been tested in clinical trials with ibrutinib for patients with CLL; however, data are available regarding the efficacy of the combination of ibrutinib and umbralisib (Supplementary Table 3).

An investigator-initiated phase II study of ibrutinib plus venetoclax was conducted for previously untreated but high-risk CLL at MD Anderson Cancer Center^76^. To reduce the risk of venetoclax-associated tumor lysis syndrome, ibrutinib alone was administered for the first three cycles for debulking. Ibrutinib plus venetoclax was then administered for an additional 24 cycles, and patients with MRD after 24 cycles continued ibrutinib alone until disease progression or unacceptable toxicities. Eighty patients initiated study treatment, and 75 patients completed the ibrutinib-only phase and initiated venetoclax. After ibrutinib monotherapy, most responses were partial, but this pattern started to change when venetoclax was added. Of the 80 patients enrolled, 59 (74%) had a CR/CRi as their best response. Twenty-six patients completed 18 cycles of therapy, and at the time of analysis, 25 of the 26 patients (96%) were in CR/CRi. After 18 cycles of ibrutinib plus venetoclax, 18 of the 26 patients (69%) were MRD-negative in bone marrow as assessed by flow cytometry. Three patients completed all 24 cycles of combined therapy; all had CR/CRi with undetectable MRD in bone marrow.

Another promising clinical trial was CAPTIVATE, an international industry-sponsored study of ibrutinib plus venetoclax in 164 treatment-naïve patients with CLL. As before^76^, ibrutinib alone was given for the first three cycles, followed by ibrutinib plus venetoclax for at least 12 cycles. After 12 cycles, according to MRD status, patients were randomized 1:1 to receive ibrutinib or placebo (undetectable MRD) or ibrutinib or ibrutinib/venetoclax (detectable MRD). In this way, the trial examined not only fixed-duration therapy but also MRD-driven personalized stopping. At 12 months of treatment with the combination, 73% of 151 patients had undetectable MRD in the blood^77^. One year after randomization, disease-free survival in patients with undetectable MRD was similar between the ibrutinib and placebo groups (*p* = 0.1475), supporting a fixed-duration treatment concept^78^.

The combination of ibrutinib and venetoclax was also tested in 80 patients with relapsed/refractory CLL. Although rates were slightly lower in this subset of patients, 68% of patients achieved undetectable MRD, demonstrating the utility of this combination in this patient group^79^.

A modified version of the ibrutinib-venetoclax combination was tested in the UK CLARITY trial^80^. In this trial, ibrutinib as a single agent was administered for 8 weeks before the combination was started. A unique feature of the study design is that duration of therapy was defined by the velocity of attaining remission and MRD response. Patients with undetectable MRD in peripheral blood and marrow at 8 months stopped ibrutinib and venetoclax at month 14. Patients who achieved a MRD-negative response later (by month 14 or 26) stopped the combination at month 26. Patients with MRD remaining at the final assessment continued ibrutinib as a single agent until progression. Fifty patients were able to complete the dose ramp-up of ibrutinib plus venetoclax. High proportions of patients achieved CR (60%) and undetectable MRD (28%) after 6 months of ibrutinib plus venetoclax. These patients were recommended to receive combined treatment until cycle 14 (12 months of ibrutinib plus venetoclax). Among 49 patients who completed cycle 14, the OR rate was 89%, and 51% of the patients had CR/CRi. Nineteen patients (36%) had undetectable MRD at the point of 12 months of combined therapy. Thirty-nine (81%) of 48 patients had no evidence of CLL according to morphological analysis of bone marrow^81^. After confirmation of MRD-negative remission, 18 patients stopped combination therapy; only one patient with undetectable MRD relapsed after treatment discontinuation by 36 months of follow-up. The disease depletion rate during the first 2 months of therapy was highly predictive of longer-term response. Patients who did not show rapid MRD eradication after 12 months had responses similar to those seen with ibrutinib monotherapy^82^.

The HOVON CLL Working Group conducted a trial of ibrutinib plus venetoclax in 51 patients with relapsed/refractory CLL. The OR rate was 96%, and 67% of the patients achieved CR/CRi after six cycles of combination therapy. The rate of undetectable MRD in blood was 29% after six cycles and 47% at the end of nine cycles^83^.

While many of the studies of ibrutinib plus venetoclax described above are at an early (1–2 years) stage of evaluation, the data are consistently and convincingly positive in all different trials. Overall, the combination of ibrutinib and venetoclax demonstrated high rates of CR and undetectable MRD for both treated and untreated CLL. At this early stage of evaluation, discontinuation of therapy appears to be highly promising as responses have been sustained.

In sum, the combination of ibrutinib with venetoclax appears promising. Results from the CAPTIVATE registration trial and GLOW (NCT03462719) randomized trial will provide definite answers. The upcoming CLL17 trial will (NCT04608318) determine whether venetoclax plus ibrutinib is better than ibrutinib monotherapy.

Roger et al. tested the triplet regimen of ibrutinib, venetoclax, and obinutuzumab in patients with previously untreated and relapsed/refractory CLL^84^. This is another example of successful time-limited trial for a total of 14 cycles. Most patients (86%) were able to complete the regimen and were eligible for response assessment. The median PFS and OS were not reached in both groups. After 14 cycles, the OR rate was 84% in treatment-naive patients and 88% in patients with relapsed/refractory disease. At the end-of-treatment assessment, the CR/CRi rate was 30% (*n* = 8) for the patients with previously untreated CLL and 44% (*n* = 11) for the patients with relapsed/refractory CLL. The proportion of patients with undetectable MRD 2 months after treatment was 28% in each cohort. Importantly, numbers of circulating NK and T cells decreased over time^84^. This phenomenon requires further investigation to determine if there is any correlation with major infections or secondary malignancies.

The ongoing ALLIANCE (NCT03737981) and ECOG-ACRIN EA9161 (NCT03701282) trials are testing the addition of venetoclax to ibrutinib and obinutuzumab for treatment-naïve patients.

Double BTK pathway blockade has not been as widely tested in clinical trials as has BTK and BCL-2 inhibition. Umbralisib is an investigational dual inhibitor of PI3Kδ and CK1ε that has recently been tested in combination with ibrutinib. Twenty-one patients with pretreated CLL were included in an investigator-initiated clinical trial of the efficacy and safety of the ibrutinib and umbralisib doublet regimen. The OR rate was 90%, and the CR rate was 29% (*n* = 6). At the 2-year time point, the PFS rate was 90%, and the OS rate was 95%. Therapy was well tolerated; no dose-limiting toxicity was observed^85^.

The triple combination of ublituximab, ibrutinib, and umbralisib was tested in a phase I dose escalation and dose expansion trial for patients with different B cell malignancies (including 23 with CLL). All CLL patients responded to therapy, and 36% had a CR; the median PFS was not reached. It is noteworthy that the rate of PI3K inhibitor-associated toxicity was low^86^.

Despite the limited data, clinical trials show that PI3K and BTK joint inhibition is feasible and warrants further investigation.

## Conclusions and future perspectives

While single-agent ibrutinib was a remarkable addition to the CLL pharmacopeia, results from combinations of ibrutinib and other agents have been even more promising. Particularly, early data from studies of the combination of ibrutinib with other targeted agents, such as venetoclax, show deep responses resulting in high CR rates and, importantly, high rates of undetectable MRD. This progress is providing us an opportunity to test fixed-duration therapy and an MRD-guided approach to discontinuation of treatment for patients with CLL. Time-limited treatment would be more cost-effective than regimens that must be used continuously until the disease progresses. MRD status and the velocity of attaining remission allow identification of patients with a low risk of relapse who do not require continuation of treatment. More selective BTK inhibitors, such as acalabrutinib, tirabrutinib, and zanubrutinib, are being tested in similar combinations. Further, the use of combination strategies to target different elements of the pathophysiology of CLL at an early stage may result in lower rates of emergence of drug-resistant clones. Long-term results will provide this long-needed information.

## Supplementary information

Supplementary Materials
