# Supplementary material for: Ibrutinib combinations in CLL therapy: scientific rationale and clinical results
Source: Blood Cancer J. 2021 Apr 29;11(4):79. doi: 10.1038/s41408-021-00467-7 (PMC8085243; doi:10.1038/s41408-021-00467-7)
Supplement: Supplementary file 1 — Supplementary Materials [file 41408_2021_467_MOESM1_ESM.pdf]

Supplementary Figure 1. Time-Line

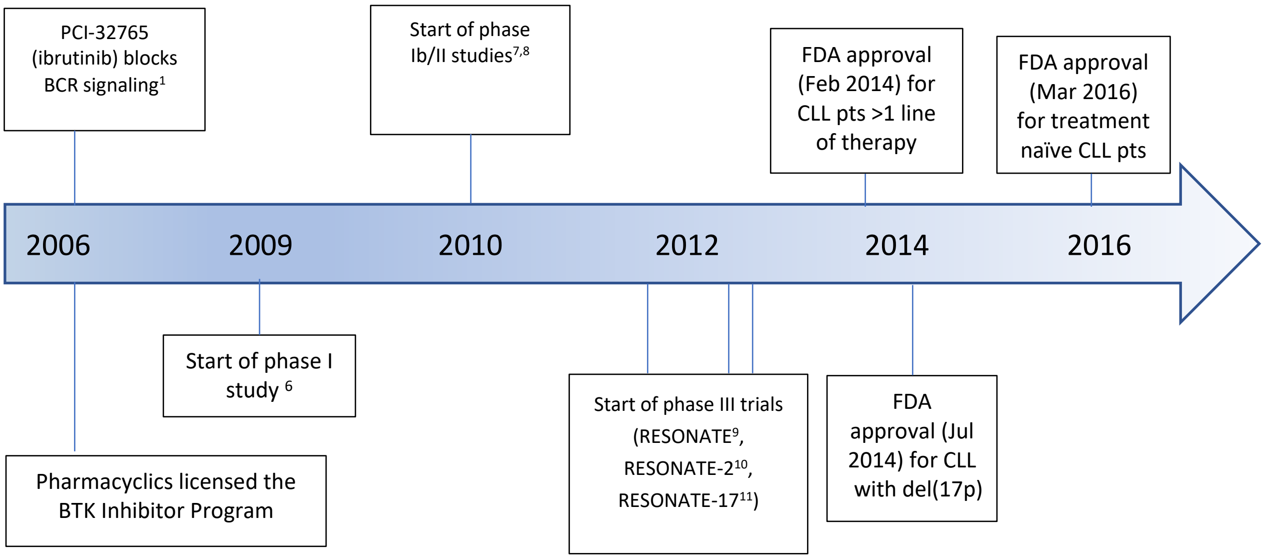

Supplemetnary Figure 1. Time-Line.
Discovery that ibrutinib blocks BTK, a key enzyme in the BCR pathway leading to phase I and II monotherapy that were responsible for the US FDA approval of ibrutinib in several subtypes of CLL; relapsed/refractory; disease with del(17p); elderly patients with CLL, and treatment naïve groups.

Supplementary Table 1. Clinical trials of combinations of ibrutinib and monoclonal antibodies in patients with CLL

Trial	Cohort	Drug(s) combined with ibrutinib	ORR and CR rate	Undetectable MRD	Safety	PFS and OS rates
Burger et al.[31]<br>NCT01520519	40 pts<br>high risk:<br>del(17p),<br>del(11q), or *TP53* mut<br>- 36 R/R<br>- 4 TN del(17p)	Rituximab	ORR – 95%<br>CR – 8%	2.6%	Pneumonia grade 2-3 – 20%<br>Infections grade 3 – 10%<br>Neutropenia grade 4 – 2.5%	18 months<br>PFS – 78%<br>OS – NR
Burger et al.[32]<br>NCT02007044	208 pts<br>- 181 R/R<br>- 27 TN with del(17p) and/or *TP53* mut	Rituximab	ORR – 92.3%<br>CR – 26%	34.4%	AE grade 3 or higher – 65%<br>Hypertension – 16%<br>Atrial fibrillation – 9.6%<br>Infections – 15.6%	36 months<br>PFS – 86.9%<br>OS – 89%
Woyach et al. [33]<br>ALLIANCE<br>NCT01886872	181 pts<br>TN	Rituximab	ORR – 94%<br>CR – 12%	BM: 4%	Hematologic AE grade 3 or higher – 39%:<br>Anemia – 6%<br>Neutropenia – 21%<br>Thrombocytopenia – 5%<br><br>Non-hematologic AE grade 3 or higher -74%:<br>Bleeding – 4%<br>Infections – 20%<br>Hypertension – 34%<br><br>Atrial fibrillation any grade – 14%, grade 3 or higher – 6%	24 months<br>PFS – 88%<br>OS – 94%
Shanafelt et al.[34]<br>ECOG-1912<br>NCT02048813	354 ots<br>TN	Rituximab	ORR – 95.8%<br>CR – 17.2%	PB: 8.3%	AEs grade 3 or higher<br>Anemia – 4.8%<br>Neutropenia – 25.6%<br>Thrombocytopenia – 4.3%<br><br>Infections – 9.4%<br>Febrile neutropenia– 2.3%<br>Atrial fibrillation– 3.2%<br>Hypertension – 18.8%	36 months<br>PFS – 89.4%<br>OS – 98.8%
Jaglowski et al.[26]<br>NCT01217749	71 pts<br>R/R, including Richter transformation (n=3), prolymphocytic lymphoma (n=2), CLL/SLL (n=66)	Ofatumumab	ORR – 83.3%<br>CR – 1.5%	Not evaluated	AEs grade 3 or higher – 59%<br>Pneumonia – 16%<br>Atrial fibrillation – 6%<br>Major bleeding event – 10%<br>1 fatal subdural hematoma	12 months<br>PFS – 83.1%<br>OS – 88.6%
Moreno et al.[35]<br>iLLUMINATE<br>NCT02264574	113 pts<br>TN	Obinutuzumab	ORR – 88%<br>CR – 19%	Overall 35%<br>BM: 20%<br>PB: 30%	Neutropenia grade 3 or higher – 18%<br>Thrombocytopenia grade 3 or higher – 15%<br>SAEs – 58%<br>1 sudden death	30 months<br>PFS – 79%<br>OS – NR
Michallet et al.[36]<br>ICLL07 FILO	135 pts<br>TN	Phase 1;<br>Obinutuzumab<br><br>Phase 2:<br>Fludarabine<br>Cyclophospha mide<br>Obinutuzumab<br>Or<br>Ibrutinib alone	ORR – 100%<br>CR – 73%	BM: 79%	AEs grade 3 or higher phase 1/ phase 2:<br>Neutropenia – 24%/23%<br>Thrombocytopenia – 32%/15%<br>Anemia – 6%/4%<br>Febrile neutropenia – 0%/4%<br><br>Gastrointestinal disorders 3%/10%<br>Cardiac events 2%/2%	24 month:<br>PFS – 98%<br>OS – 99%

Sharman et al.[37]<br>NCT 02013128	n = 41<br>R/R	Ublituximab	ORR - 90%<br>CR -5%	PB: 7%	AEs grade 3 or higher:<br>Infusion reaction – 7%<br>Anemia – 11%<br>Thrombocytopenia – 7%<br>Neutropenia – 11%<br>Diarrhoea - 4%<br>Arthralgia – 2%	NR
Sharman et al.[38]<br>GENUINE<br>NCT02301156	n=126<br>High-risk: del17p, del11q, TP53	Ublituximab	ORR - 80%<br>CR - NR	NR	Neutropenia grade 3 or higher - 7%	NR

Supplementary Table 2. Clinical trials of combinations of ibrutinib and chemoimmunotherapy

Trial	Cohort	Drug(s) combined with ibrutinib	ORR and CR rate	Undetectable MRD	Safety	PFS and OS rates
Brown et al.[53]<br>PCYC-1108<br>NCT01292135	33 pts<br>R/R<br>- 30 BR<br>- 3 FCR	Bendamustine and rituximab<br><br>or<br><br>Fludarabine, cyclophosphamide, and rituximab	BR:<br>ORR – 96.7%<br>CR – 40%<br><br>FCR:<br>ORR – 100%<br>CR – 100%	BR: NR<br><br>FCR: 66.6% (n=2)	BR:<br>AEs grade 3 or higher – 66.7%<br>Neutropenia – 40%<br>Maculopapular rash – 10%<br>Fatigue –10%<br>Thrombocytopenia – 6.7%<br>Febrile neutropenia – 6.7%<br>Cellulitis – 6.7%<br><br>FCR:<br>Gastritis with associated gastrointestinal bleeding	12 months<br>BR:<br>PFS – 86.3%.<br>OS – 84%<br><br>FCR:<br>PFS – 100%<br>OS – 100%
Chanan-Khan et al.[54, 55]<br>HELIOS<br>NCT01611090	289 pts<br>R/R	Bendamustine and rituximab	ORR – 83%<br>CR – 10%	13%	AEs grade 3 or higher – 77%<br>Neutropenia – 54%<br>Thrombocytopenia – 15%	18 months<br>PFS – 79%<br>OS – NR
Davids et al.[56]<br>NCT02251548	85 pts<br>TN	Fludarabine, cyclophosphamide, and rituximab	ORR – 98.8%<br>CR – 65.9%	83.5%	AEs grade 3 or higher:<br>Neutropenia – 35%<br>Thrombocytopenia – 32%<br>Lymphopenia – 60%<br>Febrile neutropenia – 9 %<br>Increased ALT, AST – 2%	NR
Jain et al.[57]<br>NCT02629809	45 pts<br>TN<br>IGHV mut	Fludarabine, cyclophosphamide, and obinutuzumab	ORR – 100%<br>CR – 73%	BM: 100%	AEs grade 3 or higher:<br>Neutropenia – 58%<br>Thrombocytopenia – 40%<br>Febrile neutropenia – 13%<br><br>Atrial fibrillation of any grade – 11%<br>1 death (congestive heart failure)	PFS – 97.8%<br>OS – 97.8%

Supplementary Table 3. Clinical trials of combinations of ibrutinib and targeted agents

Trial	Cohort	Combination with	ORR and CR rate	Undetectable MRD	Safety	PFS and OS rates
Jain et al.[76]<br>NCT02756897	80 pts<br>TN	Venetoclax	ORR – 100%<br>CR/CRi – 88%	61%<br>12 months	AEs grade 3 or higher – 60%<br>Atrial fibrillation – 10%<br>Hypertension – 10%	12 months<br>PFS – 98%<br>OS – 99%

	High risk and older pts [del(17p), *TP53* mut, del(11q), IGHV unmut, age ≥65 y)				Fatigue –1%<br>Myalgia – 1%<br>Diarrhea – 1%<br>Arthralgia –1%<br>Increased ALT, AST – 1%<br><br>3 cases of TLS of any grade	
Tam et al.[77]<br>CAPTIVATE<br>NCT02910583	164 pts<br>TN	Venetoclax	ORR – 97%<br>CR – NR	PB: 75%<br>BM: 72%	AEs grade 3 or higher – NR<br>AEs of any grade - 20%<br>Diarrhea – 60%<br>Neutropenia – 40%<br>Nausea – 34%<br>Upper respiratory tract infection – 24%<br>Fatigue – 20%<br>AEs leading to discontinuation – 7%	Median FU - 14.7 months<br>PFS -98%<br>OS – 100%
Jain et al.[79]<br>NCT02756897	80 pts<br>R/R	Venetoclax	NR	BM: 67%<br>24 cycles	Neutropenia grade 3 or higher – 29%<br>Thrombocytopenia grade 3 or higher– 3%<br><br>Atrial fibrillation of any grade – 9%	Median FU 22.3 months:<br>PFS – 93.75%<br>OS – 97.5%%
Hillmen et al.[80]<br>CLARITY	54 pts<br>R/R	Venetoclax	ORR – 89%<br>CR/CRi – 51%	BM: 53%<br>PB: 36%	AEs grade 3 or higher total events – 99 cases<br><br>Neutropenia – 61%<br>Thrombocytopenia – 30%<br>Lung infection – 9%<br>Hypertension – 9%<br>1 case of TLS - 2%	Median FU 21.1 months:<br>PFS – 98%<br>OS – 100%
Niemann et al.[83]<br>VISION/HOVON\ 141<br>NCT03226301	51 pts<br>R/R	Venetoclax	ORR – 96%<br>CR/CRi – 67%	29%<br>6 cycles<br>47%<br>9 cycles<br>(n=41)	NR	NR
Rogers et al. [84]<br>NCT02427451	25 TN pts<br>25 R/R pts	Venetoclax<br>Obinutuzumab	ORR 84% TN<br>ORR 88% R/R<br>CR 30% TN<br>CR 44% R/R	TN 28%<br>R/R 28%<br>2 months after 14 cycles	Hematologic AE grade 3 or higher:<br>Anemia – 2%<br>Neutropenia – 66%<br>Thrombocytopenia – 36%<br><br>Non-hematologic AE grade 3 or higher:<br>Hypertension - 38%<br>Fatigue – 8%<br>Hyperglycemia – 6%<br>Diarrhea – 6%<br>Hyperuricemia – 6%<br>Hyperkalemia – 6%<br>Upper respiratory tract infection – 4%<br>Increased AST – 4%	24 months<br>PFS – 92%<br>OS – 96%
Davids et al.[85]<br>NCT02268851	21 R/R	Umbralisib	ORR 90%<br>CR 29%	0%	Hematologic AE grade 3 or higher:<br>Anemia – 5%<br>Neutropenia – 12%<br>Thrombocytopenia – 5%<br><br>Non-hematologic AE grade 3 or higher:<br>Infections – 17%<br>Bruising – 12%<br>Diarrhea – 10%	24 months<br>PFS - 90%<br>OS - 95%
Nastoupil et al.[86]<br>NCT02006485	23 R/R	Umbralisib<br>Ublituximab	ORR- 100%<br>CR – 36%	78%	Hematologic AE grade 3 or higher:<br>Anemia – 2 %<br>Neutropenia – 22%<br>Thrombocytopenia – 7%	Median PFS – not reached<br>OS - NR

					Non-hematologic AE grade 3 or higher:<br>Cellulitis – 13%<br>Stomatitis - 7%<br>Pneumonia - 9%<br>Diarrhea – 9%<br>Hyperglycemia – 7%	

Pts – patients, R/R – relapsed/refractory, TN – treatment naïve, ORR – overall response rate, CR – complete remission, CRi – CR with incomplete bone marrow recovery, MRD – minimal residual disease, PFS – progression-free survival, OS – overall survival, PB – peripheral blood, BM – bone marrow, FU – follow-up, AEs – adverse events, TLS – tumor lysis syndrome, ALT– alanine transaminase, AST- aspartate transaminase, NR - not reported.
